# Pulmonary toxicity and lung tumorigenic potential of surrogate metal oxides in gas metal arc welding–stainless steel fume: Iron as a primary mediator versus chromium and nickel

**DOI:** 10.1371/journal.pone.0209413

**Published:** 2018-12-26

**Authors:** Lauryn M. Falcone, Aaron Erdely, Rebecca Salmen, Michael Keane, Lori Battelli, Vamsi Kodali, Lauren Bowers, Aleksandr B. Stefaniak, Michael L. Kashon, James M. Antonini, Patti C. Zeidler-Erdely

**Affiliations:** 1 Health Effects Laboratory Division, National Institute for Occupational Safety and Health, Morgantown, West Virginia, United States of America; 2 West Virginia University, School of Medicine, Morgantown, West Virginia, United States of America; 3 Respiratory Health Division, National Institute for Occupational Safety and Health, Morgantown, West Virginia, United States of America; VIT University, INDIA

## Abstract

In 2017, the International Agency for Research on Cancer classified welding fumes as “carcinogenic to humans” (Group 1). Both mild steel (MS) welding, where fumes lack carcinogenic chromium and nickel, and stainless steel (SS) increase lung cancer risk in welders; therefore, further research to better understand the toxicity of the individual metals is needed. The objectives were to (1) compare the pulmonary toxicity of chromium (as Cr(III) oxide [Cr_2_O_3_] and Cr (VI) calcium chromate [CaCrO_4_]), nickel [II] oxide (NiO), iron [III] oxide (Fe_2_O_3_), and gas metal arc welding-SS (GMAW-SS) fume; and (2) determine if these metal oxides can promote lung tumors. Lung tumor susceptible A/J mice (male, 4–5 weeks old) were exposed by oropharyngeal aspiration to vehicle, GMAW-SS fume (1.7 mg), or a low or high dose of surrogate metal oxides based on the respective weight percent of each metal in the fume: Cr_2_O_3_ + CaCrO_4_ (366 + 5 μg and 731 + 11 μg), NiO (141 and 281 μg), or Fe_2_O_3_ (1 and 2 mg). Bronchoalveolar lavage, histopathology, and lung/liver qPCR were done at 1, 7, 28, and 84 days post-aspiration. In a two-stage lung carcinogenesis model, mice were initiated with 3-methylcholanthrene (10 μg/g; intraperitoneal; 1x) or corn oil then exposed to metal oxides or vehicle (1 x/week for 5 weeks) by oropharyngeal aspiration. Lung tumors were counted at 30 weeks post-initiation. Results indicate the inflammatory potential of the metal oxides was Fe_2_O_3_ > Cr_2_O_3_ + CaCrO_4_ > NiO. Overall, the pneumotoxic effects were negligible for NiO, acute but not persistent for Cr_2_O_3_ + CaCrO_4_, and persistent for the Fe_2_O_3_ exposures. Fe_2_O_3_, but not Cr_2_O_3_ + CaCrO_4_ or NiO significantly promoted lung tumors. These results provide experimental evidence that Fe_2_O_3_ is an important mediator of welding fume toxicity and support epidemiological findings and the IARC classification.

## Introduction

Nearly half of all U.S. products require welding for their production and there are millions of welders worldwide [1]. Welding, the strongest method of joining metals, is, therefore, a common industrial practice, with gas metal arc welding (GMAW) being among the most popular modalities. In GMAW, an electric arc is established between the work piece and a consumable electrode, often mild or stainless steel (MS or SS). High temperatures create a molten pool into which the electrode is fed and the work pieces fuse as the weld cools. Inert or semi-inert gases through the welding gun serve to shield the weld from atmospheric contaminants. Arc welding processes can generate a significant amount of welding fumes, vaporized metals that condense to form particles that are respirable. The fume may contain particles from the base metal, wire/electrode, and coatings on the base metal or electrode.

Welding fumes have known adverse human health effects [1–4]. In 2017, the International Agency for Research on Cancer (IARC) classified welding fumes as a Group 1 carcinogen (*carcinogenic to humans*) [1, 5]. This classification was based on sufficient epidemiological evidence for an increased risk of lung cancer in welders even after adjusting for smoking and/or asbestos exposures that potentially confound these studies. Interestingly, positive associations were found for welding on SS, which generates fume that contain carcinogenic metals [i.e., chromium (Cr), nickel (Ni)], and MS welding where the fume is primarily iron (Fe) and manganese (Mn) [6–9]. Recent experimental animal evidence, although limited, for the carcinogenicity of welding fumes in a two-stage (initiation-promotion) model of lung carcinogenesis supported those associations [10–12]. Given the excess lung cancer risk among both SS and MS welders, despite mild persistent inflammation observed *in vivo* with SS fumes and its carcinogenic metal content compared to MS fumes [13], the degree to which the individual metals contribute to toxicity is even less clear. In a statement on welding in 2009, the IARC suggested iron fumes may be a factor and account for the excess risk [14], but epidemiology studies to date were not conclusive to evaluate specific effects attributable to iron oxides [14, 15]. Thus, experimental research needs were identified for welding fumes, a high-priority IARC carcinogen, that included evaluating the carcinogenicity of its different components [14, 16].

Welding fumes currently have no occupational exposure limit as the former Threshold Limit Value (TLV) of 5 mg/m^3^ as an 8-hour time-weighted average was retracted [17]. In the workplace, more emphasis is on regulating exposures to the most toxic metals contained in the fume (i.e., Cr or Ni). The first aim of this investigation compared the pulmonary toxicity of the primary metal oxides found in GMAW-SS fume. The second aim examined the potential of the metal oxides to function as lung tumor promoters using a two-stage initiation-promotion model in A/J mice to gain insight into the metals that drive the carcinogenicity of SS fume. Understanding the pneumotoxic effects of the individual components of welding fumes could offer a better approach for ensuring welder health and safety as sustained inflammation, oxidative stress, and persistence of the more toxic components in the lung are likely important to carcinogenesis.

## Material and methods

### Animals

Male A/J mice, 4 to 5 weeks of age, were housed in groups of two in an AAALAC International-approved specific pathogen-free, environmentally-controlled facility as previously described [18]. All animal studies were approved by the National Institute for Occupational Safety and Health (NIOSH) Institutional Animal Care and Use Committee and applicable international, national, and/or institutional guidelines for the care and use of animals were followed. Animals were acclimated to the animal facility for one week before beginning the experimental protocols and allowed access to a conventional diet (6% irradiated NIH-31 Diet, Envigo RMS, Inc., Madison, WI). All surgery was performed under sodium pentobarbital anesthesia, and all efforts were made to minimize suffering.

### GMAW-SS fume generation and metal oxide characterization

The welding fume used in this study has been fully characterized and was generated by the robotic welder designed and constructed at NIOSH as previously described [19]. The metal oxides used were Cr (as Cr(III) oxide [Cr_2_O_3_] + [Cr (VI)] as calcium chromate [CaCrO_4_], nickel [II] oxide (NiO) and iron [III] oxide (Fe_2_O_3_). Cr_2_O_3_ (product number 393703; 151.99 g/mol), CaCrO_4_ (product number CDS001277; 156.07 g/mol), NiO (product number 203882; 74.69 g/mol), and Fe_2_O_3_ (product number 310050; 159.69 g/mol) and were purchased from Sigma-Aldrich (St. Louis, MO). There is no ideal replication of the individual metal oxides formed during welding processes. GMAW-SS fume consists of chain-like aggregates of various metals and it was anticipated that the surrogate metal oxides chosen for this study would likely have a smaller diameter than the aggregates. The oxide forms of Ni and Fe presumed to be found in welding fumes are indicated above and were used as primary particles in this study. In general, Cr(VI) in shielded manual arc welding processes exists mostly in the soluble form in combination with alkali elements such as sodium or potassium often found in flux materials that protect the weld [20, 21]. GMAW-SS fumes are significantly less water soluble due to the absence of these flux materials and the Cr in these fumes is typically insoluble in the trivalent form with a very small percentage being soluble Cr(VI). Some insoluble Cr(VI) may be present in both GMAW and SMAW-SS fumes, however [20, 21]. It has been reported that the slightly soluble Cr(VI) compounds may be more carcinogenic than the highly soluble forms [22, 23]. For this reason, the Cr(VI) species (0.29% of the total fume as measured in our laboratory) tested was a slightly soluble form (CaCrO_4_) as a conservative approach.

Specific surface area (m^2^/g) of the powders was determined using nitrogen gas adsorption (ASAP 2020, Micromeritics Instrument Corporation; Norcross, GA). Cr_2_O_3_, NiO, GMAW-SS, or Fe_2_O_3_ powder was added to separate sample tubes and de-gassed under light vacuum at 300 °C for 2 hours then allowed to cool. CaCrO_4_ was degassed using the same procedure, but was held at 80 °C for 6 hrs. A value of 1.62 x 10^−19^ m^2^ was used for the molecular cross-sectional area of N_2_ at 77 °K and surface area was calculated from at least five adsorption points in the range p/p^0^ = 0.01 to 0.3. Measurements were repeated four times for each sample except for GMAW-SS, which was repeated twice. Hydrodynamic diameter and zeta potential of each study material were determined using dynamic light scattering (DLS) and laser Doppler electrophoresis, respectively (Zetasizer ZS90, Malvern Instruments, Worcestershire, UK), following dispersion in the PBS dosing medium. The pH of each sample was measured before each run using a SevenMulti calibrated electrode (Mettler-Toledo, LLC, Columbus, OH). All measurements were made at 25 °C. Parameters of the dispersant were as follows: refractive index = 1.334, viscosity = 0.9110 cP, dielectric constant = 80.2, and Henry function approximation of 1.5. Material-specific refractive index and absorbance values were used for each metal oxide. Certain metal oxides were too polydisperse for size measurement via DLS and were instead analyzed using nanoparticle tracking analysis (NTA) (NanoSight NS300, Malvern Instruments; Worcestershire, UK) to characterize mean hydrodynamic particle size (nm). For NTA analysis, the samples were injected through a Low Volume Flow Cell (LVFC) and measured at room temperature. Camera levels in the NTA instrument varied with each sample to insure accurate particle characterization. Each sample was captured 5 times for 60 seconds.

### GMAW-SS fume and metal oxide preparation

Previous metal analyses of the GMAW-SS fume have shown that the metal content of the fume is 57% Fe, 20.2% Cr, 13.8% Mn, 8.8% Ni, and 0.2% Cu [11, 19]. These metals are vaporized from the electrode/rod during welding and then condense to form the fume particles that are mainly in the form of metal oxides. However, the Cr component of this fume consists of both Cr(III) and Cr(VI), with approximately 0.29% as Cr(VI) [11]. Therefore, for this study, mice were exposed to a low or high dose of Ni as NiO, Fe as Fe_2_O_3_, and Cr as a mixture of Cr_2_O_3_ + CaCrO_4_. Mn and Cu were not investigated as they are not suspected to be carcinogenic to humans or cause lung disease. To calculate the doses in this study, the following formula was used:

$$Mass\;of\;metal\;oxide\;(mg)\;=\;\frac{\Sigma mass\;of\;metals\;(mg)}{mass\;of\;fume\;(mg)}\;\times \;(\frac{{mass\;of\;metal\;oxide}_{i}\;(mg)}{\Sigma mass\;of\;metals\;(mg)}\;)\;\times \;fume\;dose\;(mg)\times \;\frac{{Molecular\;weight\;of\;metal\;oxide}_{i}\;(\frac{mg}{mol})}{{Atomic\;weight\;of\;metal}_{i}\;(\frac{mg}{mol})}$$

The first expression in this equation indicates that the metal content is 74% of the fume mass, with the remaining mass contributed by gases including O_2_. The second expression in the equation represents the percentage of each metal (listed in the paragraph above) that contributed to that 74% of the fume mass. The third expression in the equation uses the cumulative low dose (1.7 mg) or high dose (3.4 mg) of GMAW-SS that resulted in lung tumorigenesis in our previous study [11]. The low dose of 1.7 mg represents 1.84 years of cumulative exposure and 3.4 mg represents 3.67 years assuming the previous TLV for welding fume of 5 mg/m^3^ for 8 h/d [11]. Lastly, the fourth expression in the equation represents the atomic weight ratio of the metal oxide to the metal. For example, mass (g) of NiO (low dose) = 0.74 x 0.088 x 1.7 x (74.7/58.7) = 0.141 mg.

**Fig 1:**
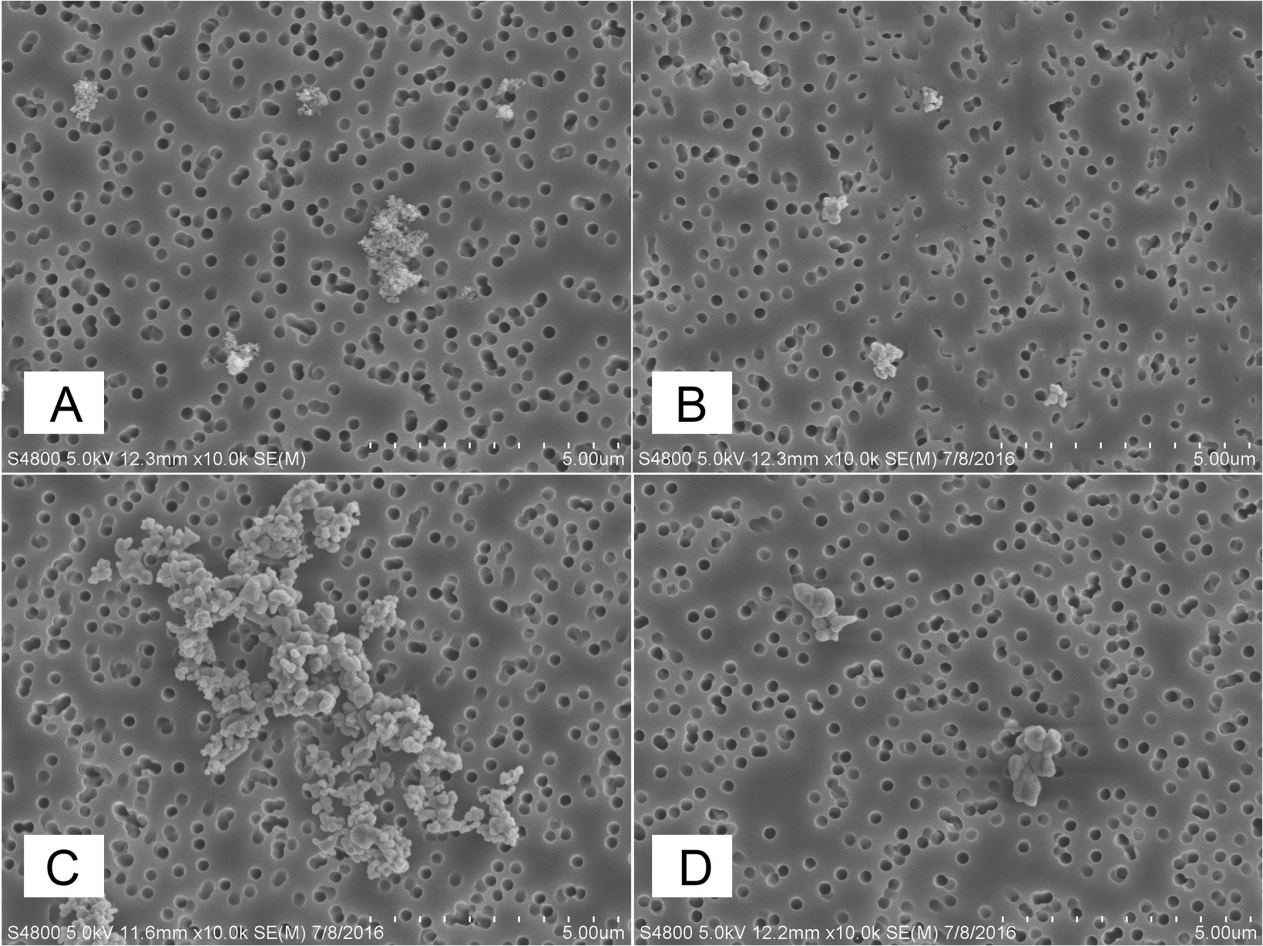
A, B, C, D. Scanning electron microscopy images of GMAW-SS (panel A), NiO (panel B), Fe_2_O_3_ (panel C), and Cr_2_O_3_ + CaCrO_4_ mixture (panel D).

The respective low and high doses of each metal oxide are shown in Table 1 and scanning electron microscopy images of GMAW-SS and the metal oxides are presented in Fig 1. The fume and each component metal were suspended in USP-grade calcium and magnesium-free phosphate buffered saline (PBS; vehicle) in a sterile conical tube. Fe_2_O_3_, GMAW-SS, and NiO were vortexed and then sonicated at 40 amps for 15 seconds using a GE 130PB ultrasonic processor (Cole Parmer; Vernon Hills, IL). The mixture of Cr_2_O_3_ + CaCrO_4_ was not sonicated.

**Table 1:** Exposure doses of the metal oxides or GMAW-SS fume.

Exposure	Low Dose	High Dose
NiO	141 μg	281 μg
Cr_2_O_3_ + CaCrO_4_	366 μg/5 μg	731 μg/11 μg
Fe_2_O_3_	1 mg	2 mg
GMAW-SS	1.7 mg	--

GMAW-SS, gas metal arc welding–stainless steel; NiO, nickel oxide; Cr_2_O_3_ + CaCrO_4_, chromium (III) oxide + calcium chromate mixture; Fe_2_O_3_, iron (III) oxide

### Mouse oropharyngeal aspiration exposure

A/J mice were exposed to the metal oxides or the GMAW-SS fume by oropharyngeal aspiration as previously described [13, 24]. In brief, each mouse was placed in a glass jar containing a histology cassette with gauze moistened with isoflurane (Abbott Laboratories; North Chicago, IL) and observed until breathing slowed. The mouse was suspended, by its front incisors, on a slanted board in a supine position. Forceps were used to extend the tongue and 50 μl of metal oxide or welding fume suspension was placed by pipette at the back of the throat. Shams received an equal volume of vehicle (PBS). The mouse aspirated the suspension into its lungs by normal breathing. The tongue was released after three deep breaths were observed. All solutions were thoroughly vortexed immediately prior to dosing. This technique loses minimal solution to the gastrointestinal tract when performed properly. The mouse was returned to its cage and resumed normal activity within 10 to 20 seconds.

### Experimental protocol 1: Biochemical measurements of lung toxicity, histopathology, and gene expression

**Fig 2:**
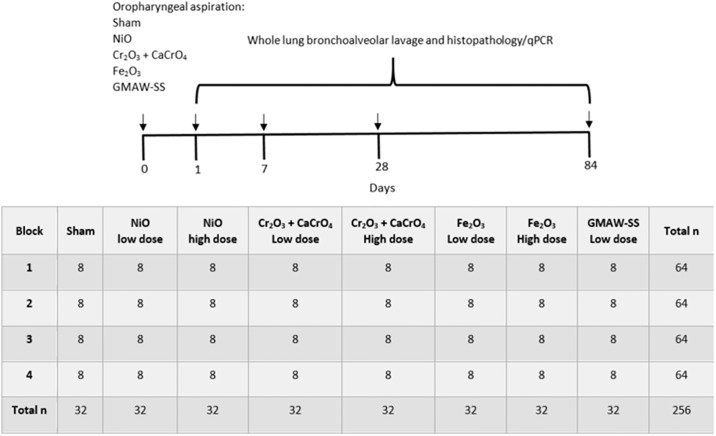
Experimental timeline and block design for experimental protocol 1: Bronchoalveolar lavage and histopathology/gene expression studies. Two groups of 256 mice were used for parallel BAL and histopathology/gene expression studies. Each group of mice was separated into 4 blocks with 8 treatment groups corresponding to the low or high doses of metal oxides, sham or GMAW-SS fume (8 groups x 8 mice/group x 4 blocks = 256 mice). Mice were sacrificed at 1, 7, 28, and 84 days after a single oropharyngeal aspiration exposure.

In two parallel studies, 256 male A/J mice were organized into 4 blocks of 64 mice and then separated into 8 treatment groups within each block (n = 8/group) consisting of a single low or high bolus dose of Cr_2_O_3_ + CaCrO_4_ mixture, NiO, Fe_2_O_3_, GMAW-SS fume (low dose only) or PBS (sham control). Doses are shown in Table 1 and a timeline of the exposure and the block design are shown in Fig 2. Animals were euthanized at 1, 7, 28, and 84 days post-oropharyngeal aspiration exposure. Mice were weighed after a weeklong acclimation period, throughout the dosing, and at the 1, 7, 28, and 84 days sacrifice. Mice were given an overdose of sodium pentobarbital (Fatal Plus; 100–300 mg/kg intraperitoneal; 390 mg/ml; Henry Schein; Dublin, Ohio) then weighed. Once unresponsive to a toe pinch, the mouse was euthanized by exsanguination.

#### Whole lung bronchoalveolar lavage (BAL) toxicity profile

A blunted cannula was placed in the trachea through a small incision and the thorax was massaged as 0.6 mL of cold PBS was instilled into the lungs. After 10 seconds, the BAL fluid was withdrawn and placed in a 15 ml conical tube. This consisted of the first lavage fraction. This process was then repeated 3 times using 1 ml of PBS per instillate and this second fraction was collected in a separate 15 ml conical tube. The BAL fluid was kept on ice and then centrifuged (500 x g, 10 minutes, 4° C).

#### Bronchoalveolar lavage fluid cytokine analysis

Cytokine concentrations from the first fraction BAL supernatant at 1 day and 28 days post-exposure were quantified simultaneously by using a Discovery Assay called the Mouse Cytokine Array/Chemokine Array 32-Plex (Eve Technologies Corp; Calgary, AB, CA). The multiplex assay was performed at Eve Technologies by using the Bio-Plex 200 system (Bio-Rad Laboratories, Inc.; Hercules, CA, USA), and a Milliplex Mouse Cytokine/Chemokine kit (Millipore; St. Charles, MO, USA) according to their protocol. The 32-plex consisted of eotaxin, granulocyte-colony stimulating factor (G-CSF), granulocyte monocyte-colony stimulating factor (GM-CSF), interferon gamma (IFNγ), interleukin-1α (IL-1α), IL-1β, IL-2, IL-3, IL-4, IL-5, IL-6, IL-7, IL-9, IL-10, IL-12 (p40), IL-12 (p70), IL-13, IL-15, IL-17, IFN-γ-inducible protein 10 (IP-10), keratinocyte chemoattractant (KC), leukemia inhibitory factor (LIF), C-X-C motif chemokine 5 (CXCL5), monocyte chemotactic protein 1 (MCP-1), macrophage- colony stimulating factor (M-CSF), monokine induced by gamma interferon (MIG), macrophage inflammatory protein 1α (MIP-1α), MIP-1β, MIP-2, regulated on activation, normal T-cell expressed and secreted (RANTES), tumor necrosis factor α (TNFα), and vascular endothelial growth factor (VEGF). Standard curves with a range of 0 to >25,000 pg/ml were determined for each cytokine. The lowest concentration in the group was used for any value that was out of range. The assay sensitivities of these markers range from 0.1–33.3 pg/ml.

#### Lactate dehydrogenase (LDH) activity and BAL cell profile

The first BAL supernatant lavage fraction was used to measure LDH activity, indicative of lung cytotoxicity. LDH activity was analyzed using a COBAS MIRA Plus auto-analyzer (Roche Diagnostic Systems; Montclair, NJ) which measured the oxidation of lactate to pyruvate coupled with the formation of NADH at 340 nm.

For analysis of the BAL cells, the supernatant from the second lavage fraction was discarded and the cell pellets of both fractions were combined. The final cell pellet suspended in 800 μl of PBS was used for cell counts and differential staining. Total cell numbers were determined using a hemocytometer. For cell differentials, cells were plated onto glass slides using a Cytospin 3 centrifuge (Shandon Life Sciences International; Cheshire, England) set at 800 rpm for 5 minutes. Slides were stained with Hema 3 Fixative and Solutions (Fisher Scientific; Pittsburgh, PA) then coverslipped. A minimum of 300 cells/slide, consisting of macrophages, lymphocytes, and polymorphonuclear leukocytes were identified using light microscopy.

#### Alveolar macrophage functional assay

To study the impact of the metal oxides on innate immune function, alveolar macrophages from the BAL cell pellet at 1, 7, and 28 days post-exposure to sham, GMAW-SS, or the metal oxides were challenged with *Escherichia coli* (*E*. *coli*) GFP for 2 hours at 1:25 MOI (multiplicity of infection) as previously described [25]. The uptake of *E*.*coli* by macrophages was quantified by flow cytometry.

#### Quantitative real-time polymerase chain reaction (qPCR) and lung histopathology

The left lung lobe was ligated, flash frozen, and then stored at -80 °C for RNA isolation while the right lung lobes were fixed in 10% neutral buffered formalin for histopathology. A liver sample was cut (~ 30 mg piece) and flash frozen before storage. RNA was isolated from the lung and liver tissues using RNeasy Mini Kits (Qiagen; Hilden, Germany) and 1 μg was reverse transcribed using random hexamers, dNTP mix and SuperScript III Reverse Transcriptase (Invitrogen, ThermoFisher Scientific; Waltham, MA). Diluted cDNA (1:10) was combined with Taqman Gene Expression Mastermix (ThermoFisher Scientific) and one of the following genes for the 1 day post-exposure lungs: heme oxygenase 1 (*Hmox1*; Mm00516005_m1) and SRY (sex determining region Y)-box 9 (*Sox9*; Mm00448840_m1). For the liver, the following genes at 1 and 7 days post-exposure were analyzed: metallothionein 1 (*Mt1*; Mm00496660_g1), metallothionein 2 (*Mt2*; Mm00809556_s1), haptoglobin (*Hp*; Mm00516884_m1), and serum amyloid A1 (*Saa1*; Mm00656927_g1). Hypoxanthine phosphoribosyltransferase (*Hprt*) was used as the reference gene (Mm03024075_m1, ThermoFisher Scientific). Amplification parameters included 10 minutes at 95°C, 1 second at 95°C and 20 seconds at 60°C. Relative mRNA levels were calculated using the comparative threshold method [26].

For histopathology studies, the right lung (consisting of apical, cardiac, azygos, and diaphragmatic lobes) was embedded in paraffin and a 5 μm standardized section was cut. Slides were stained with hematoxylin and eosin and interpreted by a board-certified veterinary pathologist in a blinded fashion. Any type or degree of lung injury and inflammation in the airways and alveolar region and evidence of changes in lung structure related to allergy including thickening around airways (epithelium and/or smooth muscle), eosinophil and lymphocyte influx, and development of bronchus-associated lymphoid tissue was evaluated. If abnormal changes were found, severity was scored as follows: 1 = minimal, 2 = mild, 3 = moderate, 4 = marked.

### Experimental protocol 2: Two-stage (initiation-promotion) lung tumor bioassay

**Fig 3:**
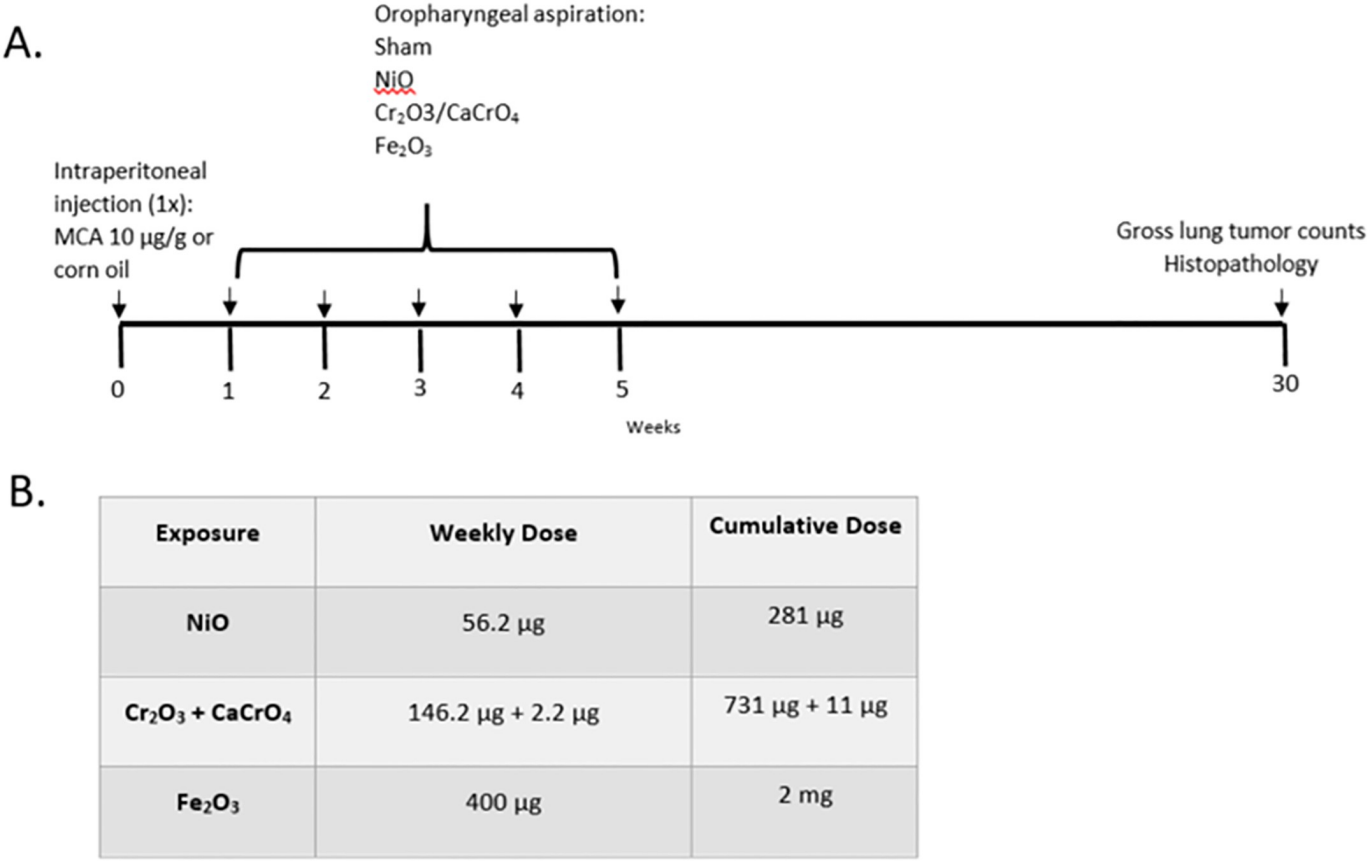
Experimental protocol 2: Two-stage initiation-promotion lung tumor bioassay. 200 male A/J mice were organized into 5 groups: MCA/NiO, MCA/Cr_2_O_3_ + CaCrO_4_, MCA/ Fe_2_O_3_, MCA/sham, or CO/sham. Beginning 1 week post-initiation with MCA or CO, mice were exposed to the metal oxide or sham by oropharyngeal aspiration once per week for 5 weeks (panel A). Doses of metal oxides were the cumulative high doses from experimental protocol 1 (panel B).

Male A/J mice (4–5 weeks of age) were organized into 5 groups (n = 40 per group) for a two stage initiation-promotion study. Mice were initiated with 3-methylcholanthrene (MCA; 10 μg/g) or corn oil (CO; vehicle control) by intraperitoneal injection (1 x). Beginning one week later, mice were exposed once per week to a mixture of Cr_2_O_3_ + CaCrO_4_ (146.2 μg + 2.2 μg), NiO (56.2 μg), Fe_2_O_3_ (400 μg), or PBS (sham control) for 5 weeks via oropharyngeal aspiration (Fig 3A). A similarly designed study was performed by our laboratory that demonstrated oropharyngeal aspiration of GMAW-SS fume significantly promoted lung tumors in mice [11]. Therefore, this exposure group was not repeated. The cumulative dose of Cr_2_O_3_ + CaCrO_4_ (731 μg + 11 μg), NiO (281 μg), Fe_2_O_3_ (2 mg) was equivalent to the bolus high dose for each metal oxide as described in experimental protocol 1 (Fig 3B). Mice were weighed after the acclimation period, throughout the dosing period, and at sacrifice.

At 30 weeks post-initiation, A/J mice were euthanized as described above. All internal organs were examined for the presence of tumors. The whole lung was then excised. The lungs were inflated and fixed with 10% neutral buffered formalin for 24 hours. Tumors were counted and measured 24 hours after fixation. Any apparent merged tumors were counted as one. Lungs were embedded in paraffin, and then a 5-μm standardized section was cut. Slides were stained with hematoxylin and eosin and interpreted by a board-certified veterinary pathologist in a blinded fashion for evidence of hyperplasia and neoplasia, inflammation, lymphoid tissue response, and foreign materials by light microscopy. Diagnostic criteria for hyperplastic and neoplastic findings were according to *go*RENI (http://www.goreni.org/), the standard reference for nomenclature and diagnostic criteria in toxicologic pathology and “INHAND,” the International Harmonization of Nomenclature and Diagnostic criteria [27, 28]. If abnormal changes were found, severity was scored by the pathologist using the following scale: 1 = minimal, 2 = mild, 3 = moderate, 4 = marked. The final severity score reflects the average of the right and left lung lobe scores and are presented as means ± standard error. Because bronchiolo-alveolar hyperplasia (BAH) and bronchiolo-alveolar adenomas (BAA) represent a continuum of the proliferative process, and there is possible overlap between these diagnoses, the number of lesions were combined to compare the tumorigenic potential of each exposure [28]. However, the gross tumor count at necropsy was more representative of the response because examination of a single histological section from a lung underestimates the total number of lesions [29].

### Statistical comparisons and analysis

Statistical analyses were done using JMP version 12 and SAS version 9.4 for Windows (SAS Institute; Cary NC). Factorial analysis of variance (ANOVA) was performed on continuous variables from the BAL fluid and the log fold changes from the qPCR analytes to make comparisons between the treatment groups. For some variables, data were log-transformed to reduce heterogeneous variance and meet the assumptions of an ANOVA. Dunnett’s test was used for individual comparisons to sham. Histopathological findings using the graded scale were analyzed using nonparametric Kruskal Wallis tests followed by Wilcoxon Rank Sum tests for pair-wise comparisons. Gross tumor counts and histopathology counts from sections were analyzed similarly. Post hoc comparisons using the nonparametric Dunn method for joint ranking were conducted to compare the treated groups to the sham group, and to compare low dose to high dose within each component. Lung tumor incidence was analyzed using a Chi-square test in SAS ‘Proc Freq,’ while tumor multiplicity was analyzed using Poisson regression in SAS ‘Proc Genmod’. In cases where overdispersion existed, a negative binomial regression was performed using data from those animals surviving to the 30-week time point. For all analyses, a *p-value* of <0.05 was set as the criteria for significance.

## Results

### Metal oxide and GMAW-SS fume characterization

Table 2 summarizes results of the material characterization. Specific surface area (SSA) of the study materials ranged from about 1 to 6 m^2^/g, consistent with their smooth micronscale compact particle morphology (Fig 1), whereas the welding fume had SSA that was at least a factor of eight higher. The higher SSA of the welding fume is consistent with its chain-like cluster particle aggregates [10]. Among study materials, hydrodynamic diameter measured following dispersion in PBS ranged from about 150 nm (chromium-containing particles) to 1000 nm (GMAW-SS). The individual surrogate metal oxides were expected to have a smaller hydrodynamic diameter compared to the total fume. For a given study material, values of hydrodynamic diameter were similar for both the high and low dose concentration suspensions, indicating that particles did not further agglomerate at the higher concentration. Values of zeta potential, which is a measure of colloidal stability, were similar among materials and for a given material did not differ between dosing concentrations.

**Table 2:** Characteristics of metal oxides and GMAW-SS fume.

Sample	Dose	SSA (m^2^/g)	Hydrodynamic Diameter (nm)	Zeta Potential (mV)**
NiO	High	2.0 ± 0.01	124 ± 0.6*	-25.8 ± 2.0
	Low		126 ± 1.1	-28.1 ± 1.7
Cr_2_O_3_ + CaCrO_4_	High	2.7 ± 0.01 + 0.78 ± 0.01	147 ± 0.3*	-28.7 ± 2.1
	Low		142 ± 2.0*	-28.4 ± 2.3
Fe_2_O_3_	High	6.0 ± 0.01	699 ± 200	-31.4 ± 0.9
	Low		639 ± 32	-32.0 ± 0.8
GMAW-SS	Low	53.1 ± 0.26	1068 ± 197	-27.1 ± 0.8

*Value determined using NTA; all other values determined using DLS

**pH of study materials: Cr_2_O_3_ + CaCrO_4_ high (7.2), low (7.3); Fe_2_O high (7.4), low (7.4); NiO high (7.4), low (7.4); GMAW-SS (6.9). GMAW-SS, gas metal arc welding–stainless steel; SSA, specific surface area; NiO, nickel oxide; Cr_2_O_3_ + CaCrO_4_, chromium (III) oxide + calcium chromate mixture; Fe_2_O_3_, iron (III) oxide

### Lung cytotoxicity after exposure to GMAW-SS or metal oxides

**Fig 4:**
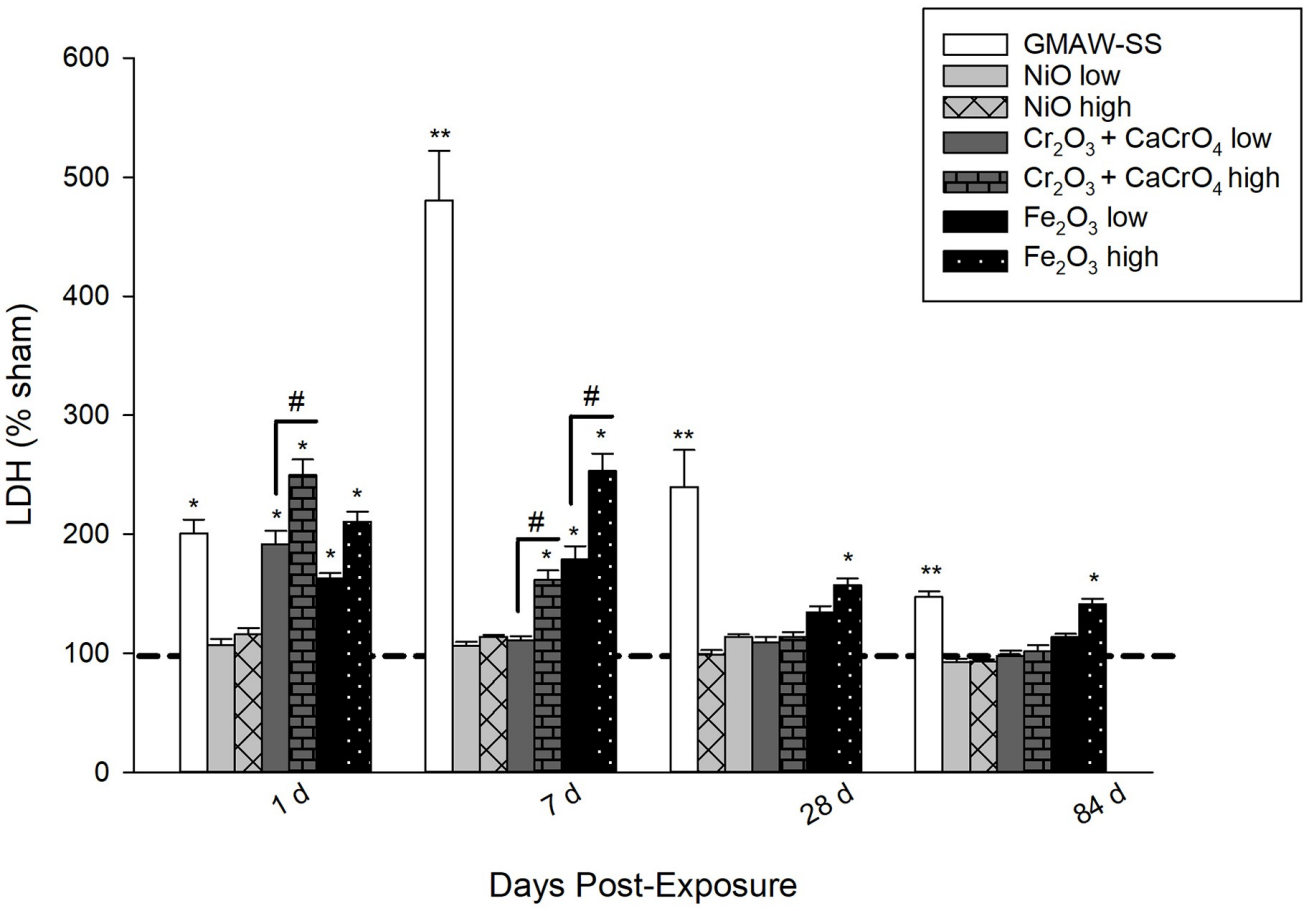
LDH activity, measured in the BAL fluid and indicative of lung cytotoxicity, after exposure to GMAW-SS fume or metal oxides. Data presented as percent change from sham (dashed line– 100%). *p<0.0001 compared to sham; **p<0.0001 compared to sham and all other low-dose groups; #*p*<0.0001 compared to low- and high-dose metal oxides within a group.

At 1 day post-exposure, BAL fluid LDH activity, indicative of lung cytotoxicity, was significantly increased in all exposed groups compared to sham except for the low- and high-dose NiO groups (Fig 4). At 7 days, it remained significantly increased in the GMAW-SS fume (~ 5 fold), Fe_2_O_3_ low and high (>2 and 3 fold, respectively), and Cr_2_O_3_ + CaCrO_4_ high-dose groups. At 28 and 84 days post-exposure, only GMAW-SS fume and Fe_2_O_3_ high-dose groups had significant lung cytotoxicity.

### BAL cell profile and macrophage function after exposure to GMAW-SS fume or metal oxides

**Fig 5:**
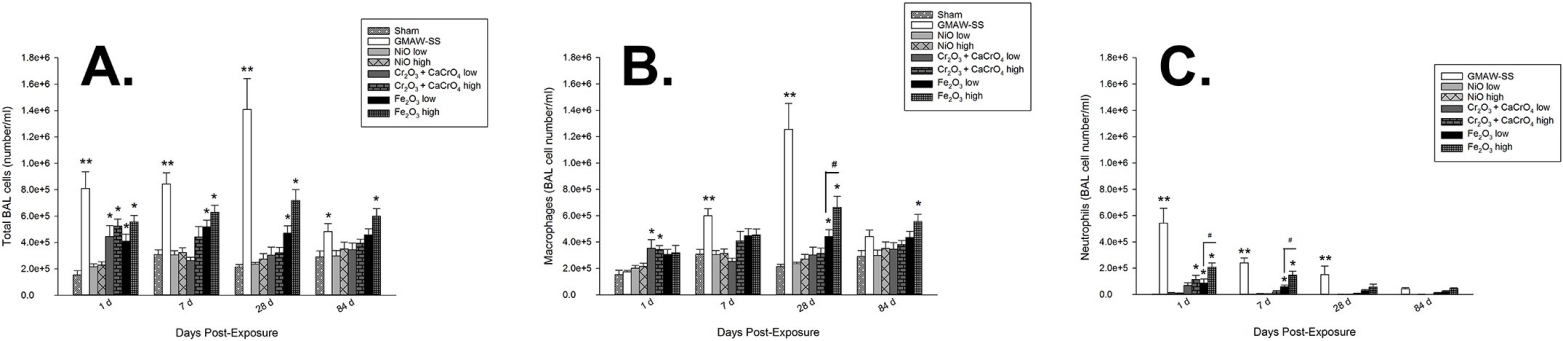
Total BAL cells (panel A), macrophages (panel B), and neutrophils (panel C) after exposure to GMAW-SS fume or metal oxides. No sham bars are present in panel c due to an absence of neutrophils in the sham group. *p<0.0001 compared to sham; **p<0.0001 compared to sham and all other low dose groups; #*p*<0.0001 compared to low- and high-dose metal oxides within a group.

**Fig 6:**
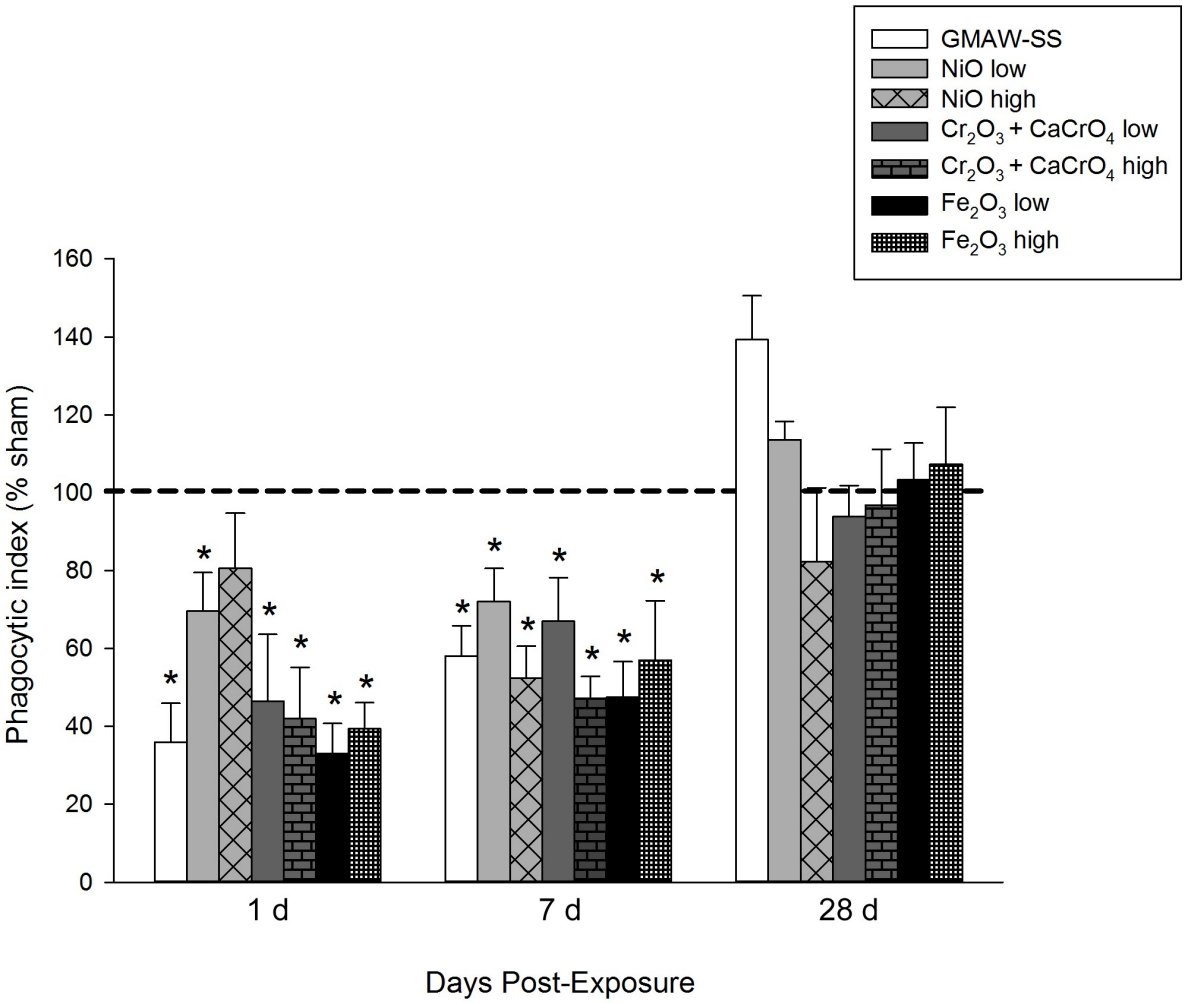
Ability of alveolar macrophages to phagocytose *E*. *coli* GFP after exposure to GMAW-SS fume or metal oxides. *E*. *coli* uptake by macrophages was quantified by flow cytometry. Data presented as percent change from sham (dashed line– 100%). *p<0.05 compared to sham.

Total BAL cells were significantly increased compared to sham in all groups except for low- and high-dose NiO at 1 day post-exposure (Fig 5A). The greatest increase in total BAL cells at 1, 7, and 28 days post-exposure was found in GMAW-SS fume-exposed mice. Significantly increased BAL cells were observed at every time point in the Fe_2_O_3_ high-dose group. The cell increases at 1 day post-exposure were mostly due to neutrophil influx, as few changes in macrophages were found at this time point (Fig 5B and 5C). However, there was significant neutrophil and macrophages in GMAW-SS fume-exposed mice at 7 and 28 days, but the macrophage influx was greater. There was a mild, but significant, eosinophil response in the GMAW-SS fume, Cr low- and high-dose, and Fe_2_O_3_ high-dose groups at 1 day post-exposure (S1 Table). By 28 days, no eosinophils were present. At 84 days, only GMAW-SS fume and Fe_2_O_3_ high-dose exposure groups had significantly increased total cells compared to sham that was primarily due to increased macrophages and some remaining neutrophils. Neutrophils were non-significant in all groups at 84 days. Macrophages were significantly increased only in the Fe_2_O_3_ high-dose group at this time point. Lymphocytes were absent in all groups throughout the time course (S1 Table). Alveolar macrophages had a reduced ability to phagocytose *E*. *coli* 1 and 7 days post-exposure to GMAW-SS fume and all component metals but returned to sham levels by 28 days post-exposure (Fig 6).

### BAL cytokine analysis at 1 and 28 days post-exposure to GMAW-SS fume or metal oxides

At 1 day post-exposure increased levels of G-CSF, GM-CSF, IL-5, IL-6, IP-10, KC, LIF, MIG, MIP-1α, MIP-1β, TNFα, and VEGF were noted in many of the exposed groups compared to sham (Table 3). Most cytokine levels were highest in GMAW-SS fume-exposed group and near sham in the NiO-exposed groups. Most cytokines resolved by 28 days post-exposure (Table 4). Fold change values from sham are shown in S2 Table.

**Table 3:** BAL cytokine analysis at 1 day post-exposure to GMAW-SS fume or surrogate metal oxides. Color scheme of yellow-orange-red represent fold change from sham (darker red represents greatest change).

	G-CSF*	GM-CSF^@^	IL-5 *	IL-6 *	IP-10 ^	KC *	LIF ^‡^	MIG ^‡^	MIP-1a*	MIP-1b*	MIP-2^#^	TNFa*	VEGF*
Sham													
NiO low													
NiO high													
Cr_2_O_3_ + CaCrO_4_ low													
Cr_2_O_3_ + CaCrO_4_ high													
Fe_2_O_3_ low													
Fe_2_O_3_ High													
GMAW-SS													

*p < 0.05 for all groups compared to sham;

^#^*p* < 0.05 for GMAW-SS fume and Cr_2_O_3_ + CaCrO_4_ and high compared to sham;

^^^*p* < 0.05 for all groups compared to sham except NiO low and high;

^‡^*p* < 0.05 for all groups except NiO low;

^@^*p* < 0.05 for GMAW-SS fume, Fe_2_O_3_ groups, and Cr_2_O_3_ + CaCrO_4_ high compared to sham

**Table 4:** BAL cytokine analysis at 28 days post-exposure to GMAW-SS fume or surrogate metal oxides. Color scheme of yellow-orange-red represent fold change from sham (darker red represents greatest change).

	G-CSF *	GM-CSF	IL-6 *	IP-10 ^#^	KC ^^^	MIP-1a ^‡^	MIP-1b ^@^	MIP-2	TNFa	VEGF
Sham										
NiO low										
NiO high										
Cr_2_O_3_ + CaCrO_4_ low										
Cr_2_O_3_ + CaCrO_4_ high										
Fe_2_O_3_ low										
Fe_2_O_3_ High										
GMAW-SS										

*p < 0.05 for GMAW-SS fume compared to sham;

^#^*p* < 0.05 for GMAW-SS fume and Fe_2_O_3_ groups compared to sham;

^^^*p* < 0.05 for GMAW-SS fume and Fe_2_O_3_ groups compared to sham,

^‡^*p* < 0.05 for all groups compared to sham except NiO low;

^@^*p* < 0.05 for GMAW-SS fume, Fe_2_O_3_ groups, and Cr_2_O_3_ + CaCrO_4_ high compared to sham

### Lung and liver gene expression analysis

Select lung and liver relative mRNA levels for genes representing systemic inflammation and oxidative stress were measured 1 and 7 days post-exposure. Recent literature suggests *Sox9* may be a new hallmark for lung adenocarcinoma so this gene was also explored in this study [30]. Expression for these genes was significantly increased following GMAW-SS fume, Fe_2_O_3_ low-dose, and Fe_2_O_3_ high-dose exposure groups at 1 day post-exposure (Table 5). By 7 days post-exposure, liver relative mRNA levels were resolving with *Mt1* increased ~3 to 4 fold in all three groups and *Saa1* increased ~3 fold and ~2 fold in GMAW-SS fume and Fe_2_O_3_ low-dose groups, respectively (S3 Table). Fewer changes in lung and liver relative mRNA levels were detected in NiO or Cr_2_O_3_ + CaCrO_4_ groups compared to the GMAW-SS fume- and Fe_2_O_3_ -exposed groups (S3 Table). No changes in relative mRNA levels occurred in NiO low- or high-dose groups in any of the genes examined. At 1 day post-exposure to Cr_2_O_3_ + CaCrO_4_ low- and high-dose, levels of liver *Mt1* and *Saa1* were significantly increased above sham (~5- and ~12-fold for low-dose and ~8- and ~17-fold for high-dose *Mt1* and *Saa1* levels, respectively; *p* < 0.05; S3 Table). At 7 days, *Mt1* and *Saa1* levels were still significantly increased ~ 2 fold in the Cr_2_O_3_ + CaCrO_4_ high-dose exposed group (S3 Table; *p* < 0.05). Among relative lung mRNA levels, only *Hmox1* levels were significantly increased in Cr_2_O_3_ + CaCrO_4_ high-dose animals compared to sham (~2-fold; p < 0.05; S3 Table).

**Table 5:** Relative mRNA levels as mean fold change ± standard error compared to sham (mean fold change of 1) in the lungs and liver at 1 day post-exposure to GMAW-SS fume or Fe_2_O_3_.

		GMAW-SS	Fe_2_O_3_ low	Fe_2_O_3_ high
**Liver genes**	*Mt1*	74.64 ± 9.02*	29.89 ± 18.16*	42.26 ± 13.46*
	*Mt2*	9.65 ± 2.58*	7.41 ± 5.03**	8.59 ± 3.23*
	*Hp*	3.93 ± 0.64*	3.24 ± 1.48*	2.31 ± 0.48*
	*Saa1*	136.57 ± 38.32*	72.02 ± 51.83*	84.34 ± 31.52*
**Lung genes**	*Hmox1*	2.69 ± 0.23*	1.54 ± 0.15**	1.92 ± 0.37**
	*Sox9*	1.63 ± 0.23	2.76 ± 0.55**	4.36 ± 1.24**

GMAW-SS, gas metal arc welding–stainless steel fume; Fe_2_O_3_, iron (III) oxide; *Mt1*, metallothionein 1; *Mt2*, metallothionein 2; *Hp*, haptoglobin; *Saa1*, serum amyloid A1; *Hmox1*, heme oxygenase 1; *Sox9*, SRY—box 9.

* *p* < 0.0001;

** *p* < 0.05

### Effects on body weight and survival post-welding fume or metal oxide exposure

Mice for experimental protocol 1 BAL group weighed on average 18.33 ± 0.13 g and for experimental protocol 1 histopathology/gene expression weighed on average 22.02 ± 0.15 g at the start of dosing. All mice survived until their respective 1, 7, 28, or 84 day sacrifices. Mice for experimental protocol 2 weighed on average 18.94 ± 0.13 g at the start of dosing and 28 of the 200 mice died prior to the planned 30 week sacrifice. All groups gained weight throughout the study and there were no significant differences found among the exposure groups *versus* sham for either protocol (data not shown).

### Lung histopathological findings for lung toxicity in A/J mice

**Fig 7:**
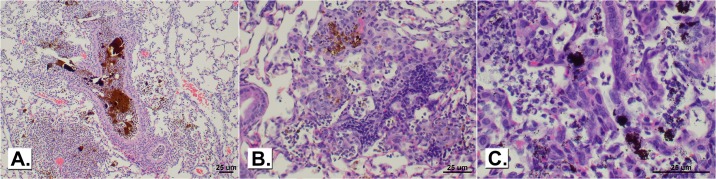
Exudate and brown material in the bronchial lumen of a mouse exposed to GMAW-SS fume and sacrificed 1 day post-exposure (panel A; 10x magnification). Mononuclear cell infiltrate of alveolar wall in a mouse exposed to GMAW-SS fume (panel B; 20x magnification) or low-dose Fe_2_O_3_ (panel C; 40x magnification) and sacrificed 7 days post-exposure.

Morphological findings are presented in Table 6 with representative photomicrographs of the findings shown in Fig 7. The response to NiO, Cr_2_O_3_ + CaCrO_4_, and Fe_2_O_3_ was characterized by the presence of black foreign bodies and pigmented macrophages in terminal bronchioles and adjacent alveoli. NiO, at 1 and 7 days, caused minimal to mild infiltration of neutrophils around terminal bronchioles and/or vessels in some instances. The response to the high-dose was not notably different from the low-dose, with the exception that black pigment and pigmented macrophages were more often detected 84 days post-exposure (S4 Table).

**Table 6:** Lung histopathology scores for the morphological findings in A/J mice at 1 day post-exposure to GMAW-SS fume or metal oxides.

	n	Infiltration, mononuclear	Infiltration, neutrophils	Pigmented macrophages	Hyperplasia	Phagocytes with cell debris	Neutrophilic exudates
Sham	6	--	--	--	--	--	--
NiO low	6	--	1.33 ± 0.42	1.00 ± 0.26	--	--	--
NiO high	6	--	1.67 ± 0.21*	1.17 ± 0.17	0.67 ± 0.33	0.33 ± 0.33	--
Cr_2_O_3_ + CaCrO_4_ low	6	1.00 ± 0.45	1.67 ± 0.21*	2.00 ± 0.00	0.17 ± 0.17	1.33 ± 0.42	--
Cr_2_O_3_ + CaCrO_4_ high	6	0.83 ± 0.40	1.33 ± 0.33	2.67 ± 0.21**	0.17 ± 0.17	1.67 ± 0.76	1.00 ± 0.52
Fe_2_O_3_ low	6	1.17 ± 0.17	1.00 ± 0.00	2.5 ± 0.22*	--	1.67 ± 0.42	--
Fe_2_O_3_ high	6	1.00 ± 0.37	1.83 ± 0.17**	3.00 ± 0.00**	0.33 ± 0.33	1.50 ± 0.50	0.50 ± 0.50
GMAW-SS	6	2.17 ± 0.17**	1.67 ± 0.21*	2.83 ± 0.17**	0.67 ± 0.42	3.17 ± 0.17**	1.83 ± 0.60

Severity scores are the averages of right lung lobes and presented as means ± standard error. Severity was scored as 1 = minimal, 2 = mild, 3 = moderate, 4 = marked.

*p < 0.05 and

** *p* < 0.005 compared to sham.

At 1 day and 7 days post-exposure to Cr_2_O_3_ + CaCrO_4_, there was minimal infiltration of neutrophils around terminal bronchioles and/or vessels and neutrophil exudate in bronchiolar and alveolar lumens in many animals, especially in the high-dose exposure group (S4 Table).

Fe_2_O_3_ caused minimal to mild infiltration of neutrophils around terminal bronchioles and/or vessels. There was neutrophil/macrophage exudate in alveolar lumens in many animals, especially in the high-dose group at 1 and 7 days. Hyperplasia of bronchiolar epithelium was rarely present. At 84 days, black foreign bodies and pigmented macrophages were consistently present in terminal bronchioles and adjacent alveolar lumens. Also, at 84 days the incidence and severity of lymphoid nodules was slightly increased (S4 Table).

The lung response to GMAW-SS fume was greater than the metal oxides and was characterized by the presence of brown foreign bodies and pigmented macrophages in terminal bronchioles and adjacent alveoli. Also, the alveolar walls were often thickened due to mononuclear cell infiltration and hyperplasia of bronchial epithelium was sometimes present at 1 and 7 days. Amorphic brown foreign bodies were occasionally present in bronchial lumens, particularly at the early time points. At 28 and 84 days, the cell response tended to transition from neutrophilic to mononuclear cell (macrophage and lymphocyte), including formation of lymphocytic nodules around vessels in affected regions. Notable lesions were still present on day 84, including pigment in terminal bronchioles that was surrounded by spindle-shaped mononuclear cells that appeared to be walling off a focal accumulation of brown pigment (S4 Table).

### Gross lung tumor multiplicity and incidence

**Fig 8:**
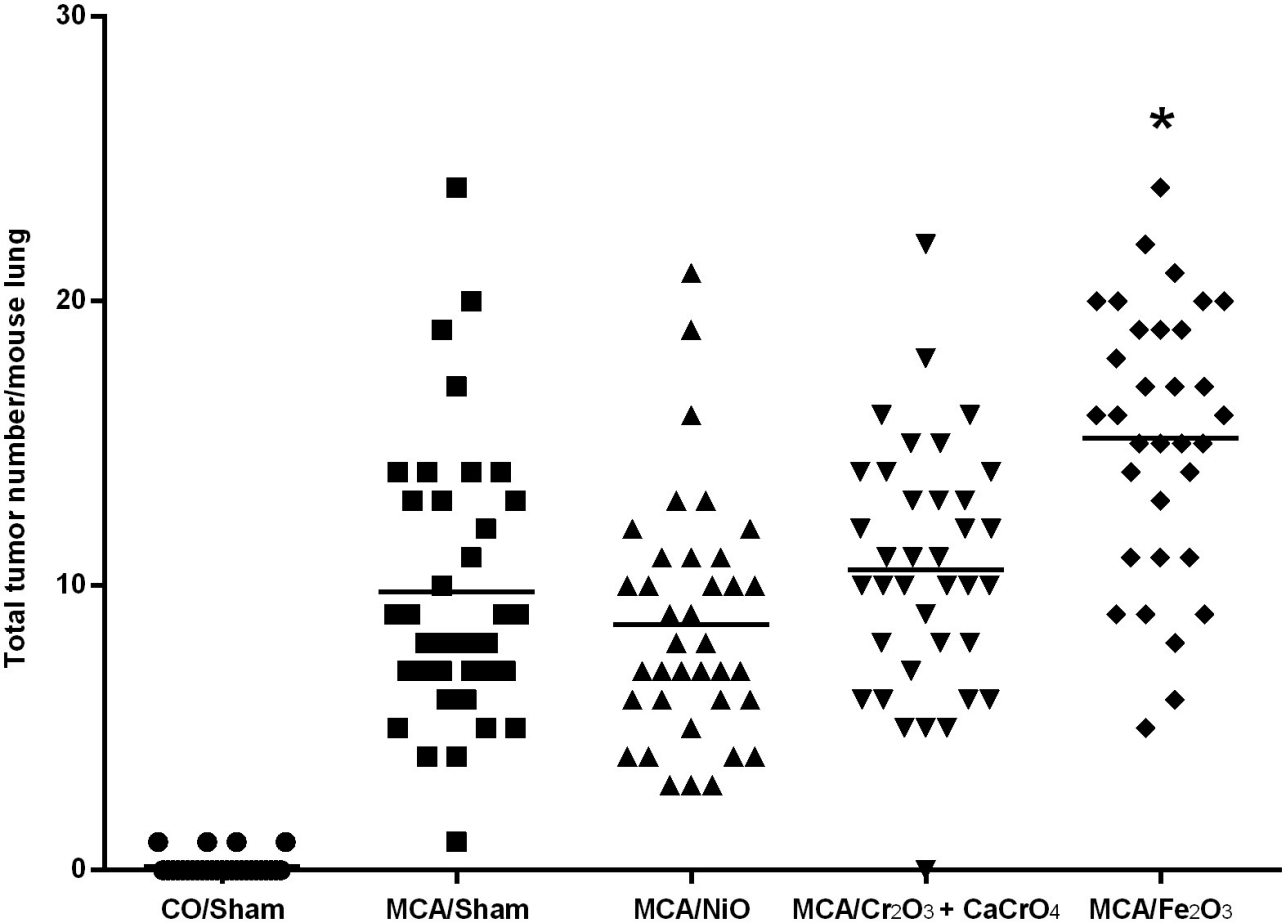
Total tumor number per mouse lung following exposure to CO or MCA and a metal oxide or sham. Bars represent lung tumor multiplicity (mean tumor number/mouse lung and includes mice with no tumors) for each group. *p<0.0001 compared to MCA/sham.

**Fig 9:**
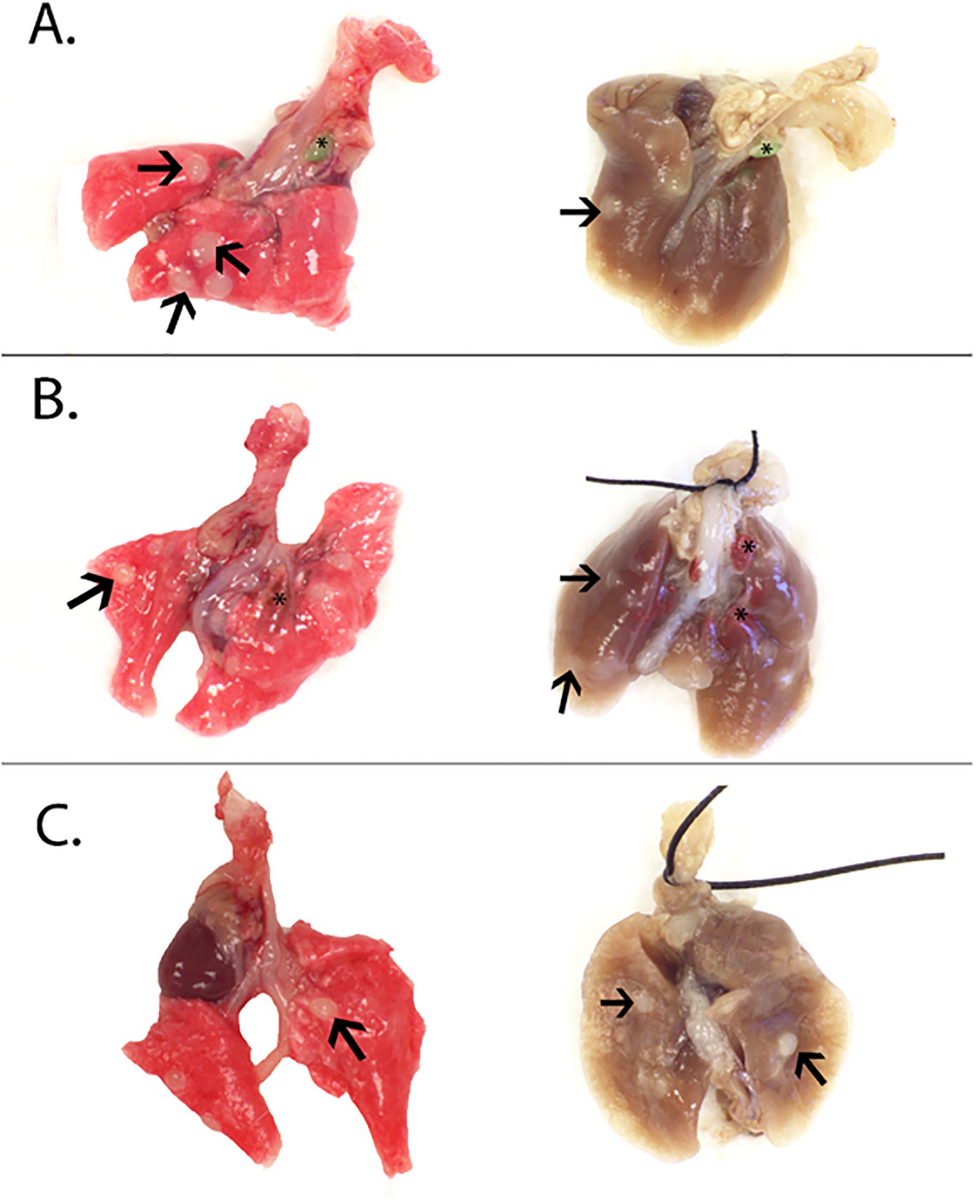
Lung tumors pre-fixation (left) and 24 hours post-fixation (right) following exposure to NiO (panel A), Cr_2_O_3_ + CaCrO_4_ (panel B), and Fe_2_O_3_ (panel C). Tumors (arrows) were on average ~1 mm in diameter and opaque in color.

Among the component metals, only Fe_2_O_3_ significantly promoted lung tumors in the A/J mouse after initiation with MCA compared to MCA/sham (15.18 ± 0.83 and 9.78 ± 0.80, respectively; *p* <0.0001). The grossly observed lung tumor multiplicity (mean tumor number/mouse lung ± SE and includes mice with no tumors) after exposure to MCA or a metal oxide is shown in Fig 8. There was no significant effect of the other metal oxides on lung tumor multiplicity compared to MCA/sham (MCA/NiO, 8.62 ± 0.69; MCA/Cr_2_O_3_ + CaCrO_4_, 10.57 ± 0.72; MCA/sham, 9.78 ± 0.80). As expected, average tumor incidence (% of tumor-bearing mice) was low in mice initiated with CO (CO/sham, 13.79%) and at or near 100% in those initiated with MCA (MCA/sham, 100%; MCA/Cr_2_O_3_ + CaCrO_4_, 97.22%; MCA/NiO, 100%; MCA/Fe_2_O_3_, 100%). Shown in Fig 9 is the gross lung morphology in sham, Cr_2_O_3_ + CaCrO_4_, NiO, and Fe_2_O_3_- exposed mice. Fe_2_O_3_ and Cr_2_O_3_ + CaCrO_4_ deposition were visible in mouse lungs and appeared red and green in color, respectively. Tumors appeared white in color and opaque on initial gross exam and became well-defined after fixation which aided enumeration. At 30 weeks, tumors were between ~0.5 and ~3 mm in diameter. Average tumor size was 1.00, 1.30, 1.17, 1.18, and 1.11 mm for CO/Sham, MCA/sham, MCA/NiO, MCA/Cr_2_O_3_ + CaCrO_4_, and MCA/Fe_2_O_3_, respectively. No significant difference in sizes among groups was found.

### Two-stage initiation-promotion lung tumor bioassay: Histopathological evaluation of lung lesions, inflammation, and presence of metals

**Fig 10:**
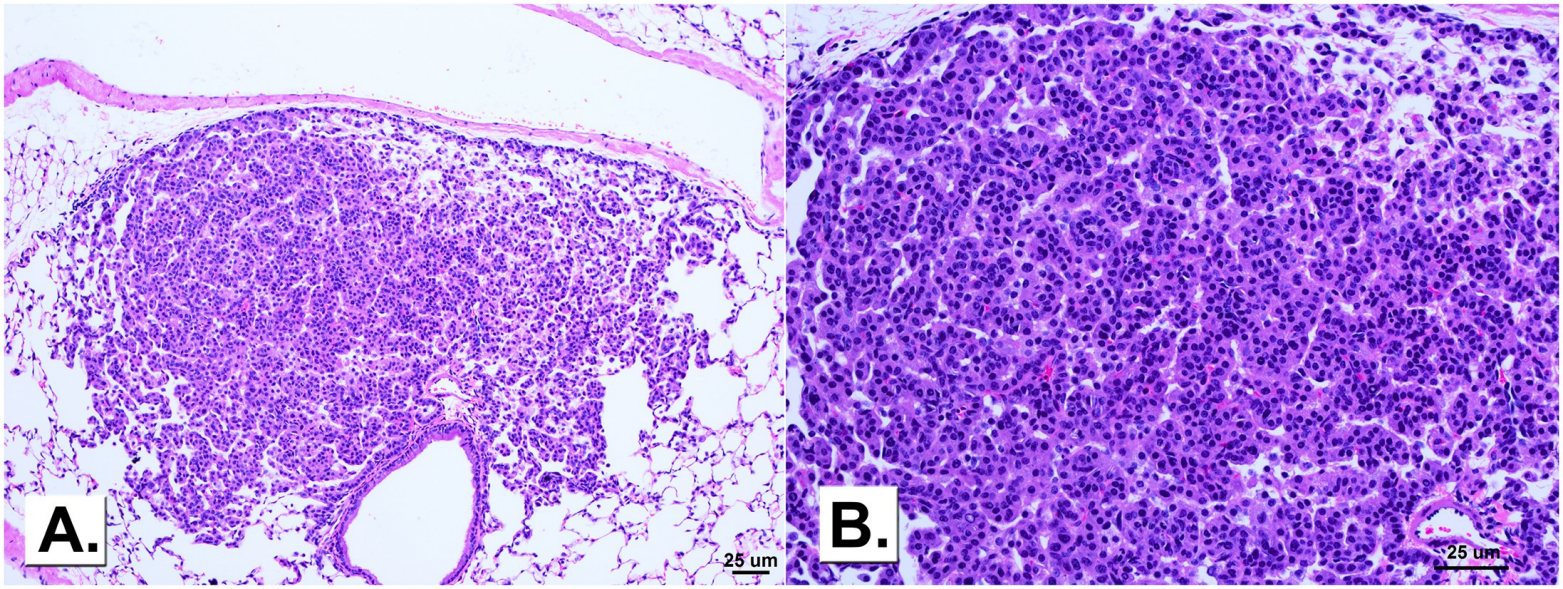
A bronchiolo-alveolar adenoma in an Fe_2_O_3_-exposed mouse 30 weeks post-initiation with MCA at 10x magnification (panel A) and 20x magnification (panel B).

Microscopic findings of the lungs of mice exposed to MCA and one of the metal oxides or sham are presented in Table 7. The most common findings were one or more bronchiolo-alveolar adenomas, and one or more foci of alveolar epithelial hyperplasia. Adenoma and hyperplasia were observed in the right and/or left lungs in all animals in all exposure groups. No carcinomas were observed. A few of the adenomas in this study were well demarcated and formed solid hypercellular masses and/or hypercellular papillary structures that had replaced the normal alveolar architecture. The adenomas were composed primarily of cells that appeared similar to those of hyperplasias, although some slightly enlarged, somewhat atypical appearing cells with enlarged nuclei were sometimes present. Mitotic figures were rare. Representative images of an adenoma are shown in Fig 10. Maintenance of the normal alveolar structure *versus* replacement by an abnormal growth pattern, is the single most important characteristic distinguishing hyperplasia from adenoma. Alveoli within larger hyperplasias generally were collapsed and appeared hypercellular, but close examination of the lesion demonstrated that the normal alveolar structure was still intact and that the lesion was a hyperplasia and not an adenoma. Alveolar epithelial hyperplasia was greatest and most severe in MCA/Fe_2_O_3_ exposed mice. Foreign material, presumably the metal, was observed in MCA/NiO, MCA/Fe_2_O_3_, and MCA/Cr_2_O_3_ + CaCrO_4_ exposure groups. The foreign material appeared as multiple, widely scattered individual or small clusters of minute discrete focal aggregates of black granules. In some cases the granules were clearly present within an alveolar histiocyte. In other cases the granules appeared to be within an alveolar histiocyte but the histiocyte was obscured by the granules. Occasionally, granules were scattered within an alveolus and not within a histiocyte. Mild lymphocytic infiltrate was observed in a few MCA/Fe_2_O_3_ and MCA/Cr_2_O_3_ + CaCrO4—exposed animals, but it was not significantly different than sham. Total lung lesions, recorded as the average number of hyperplasias and adenomas per mouse lung, were significantly increased only in the MCA/Fe_2_O3—exposed animals compared to MCA/sham (6.91 ± 0.52 *versus* 4.33 ± 0.64, respectively; *p* < 0.0001).

**Table 7:** Two stage (initiation-promotion) lung cancer bioassay: Lung histopathology severity scores for abnormal morphological findings and number of lesions in A/J mice at 30 weeks post-initiation.

	n	Lymphocytic infiltrate*	Foreign material*	Hyperplasia severity*	Alveolar epithelial Hyperplasia**	Bronchiolo-alveolar adenoma**	Total lesions**
MCA/sham	24	--	--	1.69 ± 0.30	3.00 ± 0.60 (75)	1.33 ± 0.25 (32)	4.33 ± 0.64 (107)
MCA/NiO	26	--	0.77 ± 0.17^^^	1.77 ± 0.42	2.77 ± 0.68 (72)	1.62 ± 0.48 (42)	4.38 ± 0.86 (114)
MCA/ Cr_2_O_3_ + CaCrO_4_	32	0.06 ± 0.06	0.97 ± 0.05^^^	2.19 ± 0.38	3.91 ± 0.90 (125)	1.50 ± 0.43 (48)	5.41 ± 1.02 (173)
MCA/ Fe_2_O_3_	33	0.45 ± 0.08	1.83 ± 0.05^^^	2.33 ± 0.16^+^	4.96 ± 0.44 (164)	1.94 ± 0.24 (64)	6.91 ± 0.52 (228)^^^

*Severity scores are the averages of the left and right lung lobes and presented as means ± standard error. Severity was scored as 1 = minimal, 2 = mild, 3 = moderate, 4 = marked.

**Hyperplasia and adenoma were the only two lung lesions present and represented as count data presented as average or total lesions (in parenthesis).

--Indicates no findings

^^^p < 0.0001 compared to MCA/sham

^+^p < 0.005 compared to MCA/sham

MCA: 3-methylcholanthrene

## Discussion

In this investigation, Fe_2_O_3_ was the only metal oxide to promote lung tumors in mice. At doses representative of the total GMAW-SS fume by weight percent, the lung inflammatory potential of the surrogate metal oxides was Fe_2_O_3_ > Cr_2_O_3_ + CaCrO_4_ > NiO. Overall, the pneumotoxic effects were negligible for NiO, acute but not persistent for Cr_2_O_3_ + CaCrO_4_, and persistent for the Fe_2_O_3_ exposures. Pneumotoxicity was greatest for the total GMAW-SS fume, which is consistent with other studies that reported components of the fume are less toxic than the total fume [13, 31].

The primary finding of this study was that Fe_2_O_3_ was the only metal oxide to function as a promoter *in vivo*. Iron oxides are a Group 3 or *not classifiable as to its carcinogenicity to humans* according to the IARC [32]. Occupational scenarios involving iron exposure (iron and steel founding) as well as haematite underground mining are Group 1, however, most occupational exposures to iron oxides are mixed exposures to other metals or potential carcinogens, making epidemiologic studies difficult to directly link iron oxide with a carcinogenic effect [33, 34]. In a 2009 report by the IARC, it was acknowledged that there was “an as-yet unexplained common reason” for the increased risk of lung cancer observed in several epidemiological studies with both MS and SS welding occupations [14]. Epidemiology research suggested a potential role for iron fumes, and experimental carcinogenicity assays on individual metal components of welding fumes was identified as a future research need [15, 16]. This study and more recent work showing lung tumor promotion of MS welding fumes in mice further support the epidemiological findings that MS welders, despite exposure to mainly Fe and Mn, are at increased risk of lung cancer [8, 9, 15]. Fe_2_O_3_ was the most pneumotoxic of the metals examined, as the Fe high dose group caused significant cytotoxicity and a persistent inflammatory cell response in the lung. Similarly, Fe_2_O_3_ caused the greatest increases in BAL cytokine levels, systemic inflammation, and lung histopathological alterations.

Numerous worker and animal studies have implicated Cr as contributing to lung cancer development. Cr(VI) compounds are classified as *carcinogenic to humans* (Group 1) according to the IARC and Cr(III) a Group 3 [34–38]. It has been reported that Cr(III) cannot enter cells as readily as Cr(VI) and is, therefore, less harmful [3, 36]. Cr(VI) is reduced to Cr(III) once inside cells and in the process can generate reactive oxygen species that damage DNA [36]. In the present study, the mixture of these two forms of Cr (as Cr_2_O_3_ + CaCrO_4_) did not promote lung tumors at the doses evaluated in this study but did cause mild acute cytotoxicity and inflammation at 1 and 7 days post-exposure. Levels of BAL cytokines, tissue gene expression, and lung histopathology changes were less compared to Fe_2_O_3_ and GMAW-SS fume. Nettesheim et al. 1971 performed one of the first *in vivo* Cr(VI) chronic inhalation exposure studies, exposing C57BL/6 mice, a lung tumor resistant strain, to CaCrO_4_ (13 mg/m^3^) for their lifetime and observed a significant increase in tumor incidence [39, 40]. An intratracheal instillation study in rats by Steinhoff et al. (33) reported evidence of carcinogenicity and chronic inflammation for both a soluble Cr(VI) compound and CaCrO_4_ at primarily high-dose or “irritant” levels (1.25 mg/kg for 30 months)[22]. Similarly, Glaser et al. [41] found weak evidence of carcinogenicity in a 2-year inhalation study using a soluble Cr(VI) species and a slightly soluble chromium oxide mixture (Cr_5_O_12_) in rats. Systemic effects, mild histopathological changes, and increased persistence (~10 times) of the slightly soluble Cr_5_O_12_ compared to the soluble Cr(VI) was also reported. It is apparent that high doses of Cr(VI) are needed to observe its carcinogenic effects *in vivo* [39]. Interestingly, both Steinhoff et al. and Glaser et al. suggested that chronic inflammation and/or Cr accumulation (i.e. decreased lung clearance functions) in association with a “maximally tolerated” dose may be essential for tumorigenesis. While the dose of CaCrO_4_ used in the present study (cumulative 11 μg) reflected the percentage found in the GMAW-SS fume, there was only mild acute inflammation and cytotoxicity and the dose was likely too low to result in tumorigenesis.

Like Cr(VI), Ni compounds are classified as *carcinogenic to humans* according to the IARC, with support from many worker and experimental animal studies [38, 42–45]. In 2012, the IARC concluded that high cytotoxic concentrations as well as the presence of inflammation may be needed to induce carcinogenicity [38, 46–49]. Solubility properties and speciation may play a role in carcinogenic potency of different Ni compounds with the release and accumulation of ionic Ni of seemingly high importance [38, 42, 50]. Few *in vivo* studies have specifically investigated the tumorigenic potential of NiO. Most notably, the National Toxicology Program performed 2-year chronic inhalation studies [51] that demonstrated that NiO caused inflammation and tumorigenesis in F344 rats and B6C3F1 mice. In the present study, exposure to NiO did not cause lung cytotoxicity or inflammation and the low dose, in combination with its negligible water solubility, may partially explain the absence of a tumor promoter effect.

As may be expected, GMAW-SS fume was more cytotoxic than the individual metal oxides tested. While this study demonstrated that none of the metals besides Fe_2_O_3_ functioned individually as lung tumor promoters, previous studies in our lab have demonstrated that GMAW-SS fume promotes lung tumors in A/J mice after both oropharyngeal aspiration and inhalation exposure [11, 18]. Also, histopathological changes were found in the lungs of GMAW-SS fume-exposed mice, which included both a mononuclear and neutrophilic infiltration of a greater degree compared to that of the individual metals. Changes in BAL cytokine levels as well as liver mRNA abundance were also greatest in GMAW-SS fume-exposed groups. Most notably, GMAW-SS fume increased G-CSF and IP-10 protein levels, which promote neutrophil survival and function and act as chemoattractants for macrophages, respectively, mirroring the neutrophil and macrophage influx that was observed. The greatest changes in gene expression were observed in liver at 1 day post-exposure, with increased expression of *Mt1*, *Mt2*, and *Saa1* occurring in Fe_2_O_3_- and GMAW-SS-exposed mice, indicating a potential acute phase protein response. Results from previous studies have also suggested that the total fume may be more cytotoxic than its individual components. Manual metal arc welding (MMA)-SS fume is more pneumotoxic than soluble Cr(VI), at the percent found in the fume (11). Antonini et al. (20) observed that the lung toxicity and inflammation caused by MMA-SS fume was due to both the soluble and insoluble fractions. The results of these studies, along with our findings, suggest that individual components of SS welding fumes are not capable of approximating the toxicity of the total fume. The Fe appears to be the primary component driving the persistent toxicity of the GMAW-SS fume, however, with Cr and Ni potentially contributing to the more acute effects. Further studies should determine if the sub-threshold effects of the known carcinogens, Cr and Ni, exacerbate the effects observed with Fe.

The mechanisms by which welding fumes act as lung carcinogens remain largely unknown. A recent report from Guyton et al. [52] concluded that the two most likely key carcinogenic characteristics of welding fumes include the ability to cause immunosuppression and chronic inflammation. GMAW-SS fume has been shown to cause sustained cellular influx in rodent models and this study found that both GMAW-SS fume and Fe_2_O_3_ were pneumotoxic with local and systemic inflammatory responses observed after a single bolus dose [13, 53, 54]. Epidemiology studies indicate that welders and metal fume workers are more susceptible to develop an infection [55–58]. In agreement, inhalation to GMAW-SS fume suppressed the lung’s ability to clear *Listeria monocytogenes* in Sprague-Dawley rats [53]. The present study also demonstrated that the metal oxides and GMAW-SS fume inhibited the ability of macrophages to phagocytose bacteria, suggesting that an immunosuppressive state was induced but alone was not sufficient for tumor promotion. Alterations in phagocytic function has been shown to be caused by an alteration in surface receptors with a phenotypic change in alveolar macrophages [59, 60]. These mechanisms, likely in combination, potentially contribute to tumorigenesis.

There are a number of limitations to this study. First, the metal oxides used for exposure were pure oxides and were not isolated directly from the fume. The metals in the freshly generated fume are more complex and may consist of different chemical compositions and combinations of these oxides; therefore, the oxides in the fume may differ in their morphology, reactivity, and solubility compared to those used in this study. For this reason, we characterized the metal oxides used in this study and noted that each oxide had much smaller SSA and different hydrodynamic diameters than the GMAW-SS. A second limitation to this study was that the exposures were done by oropharyngeal aspiration of bolus doses. Although this is a well-established method and both inhalation and oropharyngeal aspiration of this welding fume promoted lung tumors [10, 11], the bolus doses are less representative of worker inhalation exposures. A further limitation of this study was that metals were dosed individually and not in combination, which eliminated the examination of any potential additive or synergistic effects among two or more metal oxides.

In summary, the results of this study provide experimental insight into the toxicity and tumorigenicity of some of the abundant metal oxides found in welding fumes. In particular, it was found that Fe_2_O_3_ is a lung tumor promoter *in vivo* and may be the primary metal oxide responsible for the carcinogenic effect of SS fume. The results also add quantitative support to an earlier hypothesis from the IARC that indicated a common reason for excess lung cancer risks for all welders beyond Ni and Cr(VI) given that welding processes not associated with carcinogenic metals are now classified as a Group 1 carcinogen [5, 14]. Additional studies will focus on further investigating the toxicity of these metal oxides *in vivo* and *in vitro* and exploring potential mechanisms of tumorigenesis of different welding fumes and combinations of the metal components.

## Supporting information

S1 Table (XLSX)

S2 Table (XLSX)

S3 Table (XLSX)

S4 Table (XLS)
